# The prevalence of developmental defects of enamel, a prospective cohort study of adolescents in Western Sweden: a Barn I TAnadvarden (BITA, children in dental care) study

**DOI:** 10.1007/s40368-018-0347-7

**Published:** 2018-05-14

**Authors:** B. Jälevik, A. Szigyarto-Matei, A. Robertson

**Affiliations:** 1 Department of Pediatric Dentistry, Institute of Odontology at the Sahlgrenska Academy, University of Gothenburg, Gothenburg, Sweden; 2 Clinic of Pediatric Dentistry, Public Dental Service, VGR, Uddevalla, Sweden

**Keywords:** Developmental defects of the enamel, Epidemiology, Aesthetics

## Abstract

**Aim:**

To describe the prevalence of different types of developmental defects of the enamel (DDE) in varying age-cohorts and habitations, and to analyse if early trauma to the primary teeth and early subsequent serious health problems were related to DDE in the permanent dentition. Dental fear and anxiety, and aesthetic problems as a consequence of DDE were also investigated.

**Methods:**

DDE was registered over 5 years annually in three age cohorts (796 children). The DDE index (FDI Commision on Oral Health, Research and Epidemiology, Int Dent J 42:411–426, 1992) was used. Information on diseases in early childhood, trauma to the primary teeth, and dental fear and anxiety were collected.

**Results:**

The prevalence of DDE was 33.2% (boys 37.1%, girls 29.3%, p = 0.02). Demarcated opacities (DEO), solely, were the most frequent kind of defect, affecting 18%. Five percent (5%) had diffuse opacities (DIO) and 1% had hypoplasias, whereas 7% had teeth with both DEO and DIO. The most frequently affected teeth of DEO, as well as of DIO, were the first permanent molars and maxillary central incisors. Dental injuries to the primary anterior teeth raised the risk for DDE in the permanent teeth, but early serious health problems did not. Generalised DDE was common (8.4%). The paediatric dentists assessed the DDE in the maxillary anterior teeth as more serious than did the affected children and their parents.

**Conclusions:**

Generalised DDE was more frequent than expected, as well as the occurrence of both DEO and DIO in the same individual. The first permanent molars and the upper central incisors were the most affected teeth.

## Introduction

Developmental defects of enamel (DDE) are common. As high as 50% of a population, living in a low fluoride district, has been shown to have at least one tooth with mineralisation disturbances. The fluoride content of drinking water has a considerable effect on the DDE prevalence (Dummer et al. 1990).

DDE teeth can be problematic for the affected child, as well as the treating dentist, with considerable aesthetic problems ensuing. The teeth also may break down and be impaired by shooting pain and difficulties in anaesthetising. Dental fear and anxiety, as well as dental behaviour problems as a consequence of DDE, have been reported (Jälevik and Klingberg 2002).

For the dental staff, sound knowledge, early recognition, and treatment planning of DDE are of the utmost importance for optimal care and the prevention of dental fear.

Developmental defects in the enamel are defined as disturbances in hard tissue matrices and in their mineralisation, arising during odontogenesis (Commission on Oral Health, Research and Epidemiology, FDI 1992). The defects may be localised, affecting single or multiple teeth, or systematic, affecting groups of teeth developing at the time of disturbance (Commission on Oral Health, Research and Epidemiology, FDI 1992).

### Nomenclature

Historically, a wide variety of terms and definitions have been used to describe developmental defects of the enamel. Some are simply descriptive terms while others are linked to the causative agent, e.g., fluoride. To remedy this confusion, an FDI working group was established in 1982 and the DDE index was published. This original index was complicated to use in practice and a modified DDE index (mDDE) was presented in 1992. In short, DDE is classified as demarcated opacities, diffuse opacities, and hypoplasia.

*Hypoplasia* is defined as a quantitative defect of enamel involving the surface, with reduced thickness of the enamel. The defective enamel may occur as shallow or deep pits or rows of pits arranged horizontally, or as small or large, wide or narrow grooves.

*Opacity* is defined as a qualitative defect of enamel identified, i.e., as an enamel hypomineralisation; visually as an abnormality in the translucency of enamel. A white or discoloured area is characteristic, but the enamel surface is smooth and its thickness is normal. There are two kinds of opacities; *demarcated opacity* with a distinct and clear boundary to the adjacent normal enamel and can be white, cream, yellow or brown in colour; *diffuse opacity* has a linear, patchy or confluent distribution, but there is no clear boundary to the adjacent normal enamel.

The examination prerequisites were given: tooth surfaces were inspected visually and defective areas tactilely explored with a probe. Natural or artificial light was used during examination and defects less than 1 mm were not recorded. The teeth were not dried, but large debris was removed with the help of a cotton roll. The number of subjects with one or more teeth affected, the mean number of teeth per child affected by any defect, and the type of defect were the standard data to be reported (Commission on Oral Health, Research and Epidemiology, FDI 1992).

In the twenty-first century, studies of DDE have mainly focused on MIH. The mDDE index was considered to be too time-consuming and not adequate for MIH studies, as post-eruptive breakdown (PEB) is a prominent feature in MIH and the mDDE index may not clearly differentiate PEB from hypoplasia. In addition, the mDDE does not represent atypical restorations and extractions owing to MIH. In the EAPD seminar in 2003, specific criteria for MIH in epidemiological studies were established (Weerheim et al. 2003).

### Prevalence

The relationship between dental fluorosis and the content of fluoride in drinking water is well-documented since a long time ago (Dean 1934; Møller 1982). However, the interest in studying the presence of opacities in low fluoride areas has historically been low (Small and Murray 1978). Moreover, before the DDE indexes were published, there was great confusion concerning the description and nomenclature of enamel defects, making earlier studies of enamel opacities hard to interpret.

Studies on full mouth examinations of the permanent dentition, using the mDDE-index, are infrequent. Clarkson and O’Mullane (1992) showed that 63% of 15-year-olds in a low-fluoride district had at least one tooth with DDE and that demarcated opacities (DEO) were predominant. Seow (2011) found that 58% of a study group, with permanent teeth and a mean age 13.5 years, had at least one tooth affected by DDE, and that DEO and DIO were equally represented in the permanent dentition. Robies et al. (2013) examined children 3–12 years old; 52% of those with only permanent teeth, had at least one tooth affected. DEO was the most frequent defect. A prior prevalence study from the region of the present survey found that 33% of 8-year-olds had at least one tooth affected (Jälevik et al. 2001).

In the past decades, a number of prevalence studies of MIH have been published from different parts of the world. A wide variation in MIH prevalence has been reported (Jälevik 2010). In spite of the EAPD criteria from 2003, cross-comparisons of the results from these various studies have been difficult due to the use of different indices and criteria, examination variability, methods of recording, and varying age groups (Jälevik 2010).

### Aetiology

Defects can be genetic or environmental and often the cause remains unknown. Environmental (also called acquired) dental defects can be divided into those caused by local factors and those caused by systemic factors. A local factor can be suspected when a single tooth or group of neighbouring teeth is affected. General symmetric defects, related to the timing of the insult and thus to the sequence of the development of the teeth, are denominating chronological defects. The aetiology of general defects, not related to any particular time period during tooth formation (non-chronological defects), are either genetic or due to non-genetic, longstanding environmental influences, e.g., intoxications, malnutrition or other medical conditions (Espelid et al. 2017; Wong 2014).

Well-known enamel defects are amelogenesis imperfecta, dental fluorosis, enamel defects in permanent teeth caused by trauma or infection to the preceding primary teeth, and MIH.

*Amelogenesis imperfecta* (AI) is a genetically determined developmental defect of the enamel, affecting all or the majority of teeth in the dentitions. The enamel defects are in general more pronounced in the permanent dentition, compared to the primary dentition (Crawford et al. 2007). The reported prevalence of AI varies from 0.06/1000 in the USA, to 0.25/1000 in Southern Sweden and 1.4/1000 in Northern Sweden (Witkop and Sauk 1976; Sundell and Koch 1985; Bäckman and Holm 1986).

*Dental fluorosis* is a well-known, environmental developmental defect of the enamel. The causal connection between fluorosis and percentage of fluoride in drinking water is well-documented. Diffuse opacities affecting homologous teeth are characteristics of fluorosis. The opacities vary from fine white transverse lines to extensive opaque areas. The border to normal enamel is always diffuse. Post-eruptive staining and shallow pits are common in more serious cases (Möller 1982; Cutress and Suckling 1990; Fejerskov et al. 1996).

*Trauma* to the primary incisors is the most well-documented local causative factor for DDE affecting the permanent successor. It has been shown that about 10% of enamel defects in permanent incisors have been caused by trauma to the preceding incisor. Trauma at a very early age involves a greater risk of hyperplastic defects, compared to later trauma that can cause opacities in the permanent successor (Andreasen et al. 1971). An infected primary tooth may also increase the risk of DDE in the permanent successor (Turner’s teeth) (Turner 1912).

*Molar incisor mineralisation* (MIH) is an acquired, systemic enamel defect in the permanent first molars (PFM) (Weerheim et al. 2001) and is frequent in many populations (Jälevik 2010). Approximately every fifth child in Sweden has been shown to have MIH, often in combination with post-eruptive enamel disintegration. One, two, three or all PFM could be affected to some extent. One or more of the incisors are often affected at the same time. (Jälevik et al. 2001). The causation of MIH is still not confirmed.

### Aesthetic perception of DDE

Few studies have considered aesthetic problems owing to DDE, with the appearance of fluorosis mostly discussed. Chankanka et al. (2010) showed that mild fluorosis was not associated with negative effects on Oral Health Related Quality of Life (OHRQoL), but severe fluorosis was consistently reported to have negative effects on OHRQoL. In another study (McGrady et al. 2012), demarcated opacities in the incisor were judged to be more troublesome than mild fluorosis. Sujak et al. (2004) investigated the affect of all types of DDE. They suggested that very few subjects were concerned about the appearance of their teeth, or were not aware of their teeth being different.

The aim of the present study was to investigate:

- The prevalence of developmental defects (DDE) in the enamel of permanent teeth in varying age cohorts and habitations.
- The possibility of revealing diagnoses with the help of mDDE-index.
- The connection with early childhood traumatic injuries in the primary dentition and DDE.
- The connection with serious health problems in early childhood and DDE.
- Dental fear and anxiety in children/adolescents with DDE.
- The aesthetic perception of DDE judged by children/adolescents, their caretakers, and paediatric dentists.

## Materials and methods

The BITA study (BITA = Barn I TAndvården, which means children in dental care) is a 5-year longitudinal study in Sweden, between the years 2008 and 2012, concerning different aspects of children in the dental situation. Four age cohorts with children 3, 7, 11 and 15 years at the study start, from five Public Dental Service clinics in the Region Västra Götaland, were invited to participate, representing both rural and urban areas with different socio-economic status. The majority of the patients had municipal drinking water with a low level of fluoride (< 0.10 mg/ml). In the more rural areas, some had drinking water from a well, but there was no accessible information on the fluoride status of any private drinking water supplies.

Developmental defects of the enamel in all erupted permanent teeth were registered yearly for 5 years, discerning between demarcated opacities, diffuse opacities, and hypoplasias, according to the mDDE-index (FDI 1992). Moreover, post-eruptive breakdown of the enamel in affected teeth was recorded. The youngest cohort was excluded from the DDE part of the BITA study as the children were 7 years old by the study end and consequently, only had a few permanent teeth to examine.

After instruction and training, the ordinary dental staff carried out the examinations. A test protocol, composed of 24 digital photos of teeth with DDE, completed; 85% of the affected surfaces were correctly assessed. The study cohorts were 11, 15 and 19 years old at study end.

Data from the clinical examination concerning type and distribution of DDE were analysed. Annual background data on traumatic injuries of the primary teeth 0–6 years of age, severe illness during the first 4 years of life, and dental fear and anxiety, were collected from the BITA study. The CFSS-DS scale measured dental fear and anxiety.

At the study end, information on fluoride in drinking water and known family enamel defects were collected from those with registered DDE, in a questionnaire. The children/adolescents, as well as the caretakers, were also asked for aesthetic and treatment problems caused by their enamel developmental disturbances.

Information on treatment measurements of the permanent first molars was collected from the record of all subjects with registered defects.

Ninety-six randomly selected children/adolescents, with observed DDE, were clinically examined by two paediatric dentists (BJ and AM) in order to verify the registrations. Intraoral photos were taken. The aesthetic appearance on the photos of the front teeth (canines and incisors) was assessed by the paediatric dentists. That assessment was compared to reported aesthetic problems for the patients and their caretakers.

### Analysis

Data were entered into a spreadsheet (Microsoft Excel, Microsoft Corp, WA, USA) and then analysed using SPSS Version 22, (SPSS Inc, Chicago IL, USA). Descriptive statistics were used to assess the participants data. Responses to questionnaires were analysed through the use of descriptive statistics, and comparisons between different dental groups were made with the Chi square test and odds ratio. Concerning CFSS-DS, the T-test was used. Results at an alpha level less than 0.05 were considered statistically significant.

### Ethical considerations

#### BITA-study

Application for an ethical review (Dnr: 286-07) was submitted in June 2007. The Regional Ethical Review Board of the University of Gothenburg, Sweden, found the project was not subject to the Swedish Act on Ethical Review. The Board provided feedback on the information to the patients, which was taken into account during further planning of the study. The caretakers and their children received a letter with information regarding the project before entering the BITA-study. Respondents were asked to participate in the study when they attended a routine dental appointment. In connection to the first clinical examination, the caretakers signed an informed consent to participate in the research project.

#### Questionnaire

The study was approved by the Regional Ethical Review Board of the University of Gothenburg, Sweden, Dnr: 806-13 (2014-02-25). Children/adolescents and their caretakers were given written information regarding the study and asked to give their consent to participate.

## Results

At the BITA-study start, 964 children were eligible; 168 left the study before the study end and 796 children (83%) remained. The reasons for dropping out were mainly moving to another location, parents’ lack of time, unwillingness to fill in questionnaires and non-appearance.

The subsequent questionnaires were completed by 201 of 264 (76%) participants having developmental defects of the enamel (DDE).

### Prevalence of DDE

**Fig. 1:**
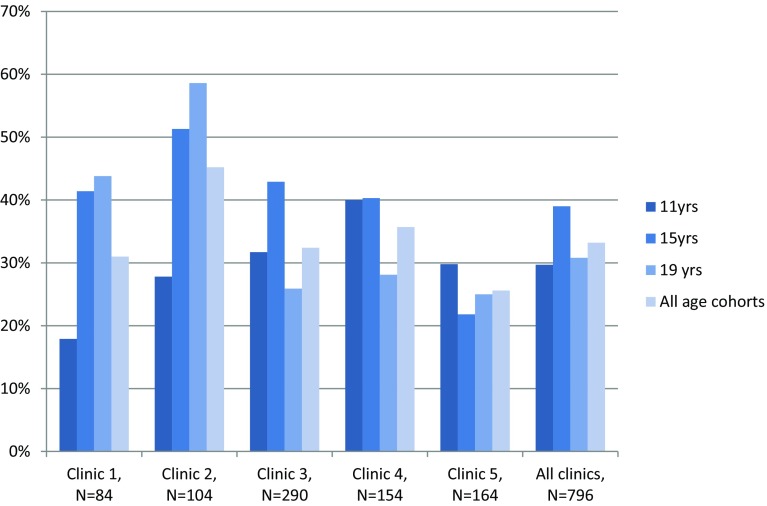
The distribution of developmental defects of the enamel (DDE) among participating clinics and the age cohorts

The distribution of gender was even in the study population. Developmental defects (DDE) were registered in 264 (33.2%) children. More boys than girls had DDE (Table 1). DDE was more prevalent in the 15-year-olds (Cohort 3), compared to the other age cohorts (p = 0.05) (Table 2). The prevalence in the participating five clinics varied from 25.6 to 45.2% (p = 0.02). The most rural clinic had the highest prevalence (Fig. 1).

**Table 1:** The distribution of developmental defects of the enamel (DDE) by gender

	DDE		Total
	No	Yes	
	N (%)	N (%)	
Girls	283 (70.8)	117 (29.3)	400
Boys	249 (62.9)	147 (37.1)	396
Total	532 (66.8)	264 (33.2)	796

The boys were significantly more affected than the girls, p = 0.02

**Table 2:** The distribution of developmental defects of the enamel (DDE) among the age cohorts

Age	DDE		MIH		General. DDE		Total, N
	No, N (%)	Yes, N (%)	No, N (%)	Yes, N (%)	No, N (%)	Yes, N (%)	
Cohort							
11	185 (70.3%)	78 (29.7%)	218 (82.9%)	45 (17.1%)	262 (99.6%)	1 (0.4%)	263
15	163 (61.0%)	104 (39.0%)	237(88.8%)	30 (11.2%)	235 (88.0%)	32 (12.0%)	267
19	184 (69.2%)	82 (30.8%)	244 (91.7%)	22 (8.3%)	232 (87.2%)	34 (12.8%)	266
Total	532 (66.8%)	264 (33.2%)	699 (87.8%)	97 (12.2%)	729 (91.6%)	67 (8.4%)	796
	p = 0.05		p = 0.01		p < 0.001		

The table also shows the distribution of MIH and generalised DDE judged from the clinical registration and information in the dental records

### Type of DDE

Half of those with DDE had solely demarcated opacities (DEO). However, fully one-quarter had teeth with DEO, as well as one or more teeth with diffuse opacities (DIO) and/or hypoplasias (Hypo). The most frequent “mixed DDE” was DEO + DIO, affecting more than one-fifth of those with DDE (Table 3).

**Table 3:** The distribution of the different types of developmental defects of the enamel (DDE) among the affected dentitions

DDE type	N	% affected (264)	% total sample (796)
DEO	144	54.5	18.1
DIO	42	15.9	5.3
Hypo	11	4.2	1.4
DEO + DIO	54	20.5	6.8
DEO + Hypo	7	2.7	0.9
DIO + Hypo	1	0.4	0.1
DEO + DIO + Hypo	5	1.9	0.6

*DEO* demarcated opacities, *DIO* diffuse opacities, *Hypo* hypoplasias

Post-eruptive breakdown (PEB) of the enamel was registered in 19.3% of the subjects with DDE. PEB was more common in subjects with one or more teeth with demarcated opacities (21.4%), compared to those with diffuse opacities and/or hypoplasias (11.1%).

### Distribution within the dentition

**Fig. 2:**
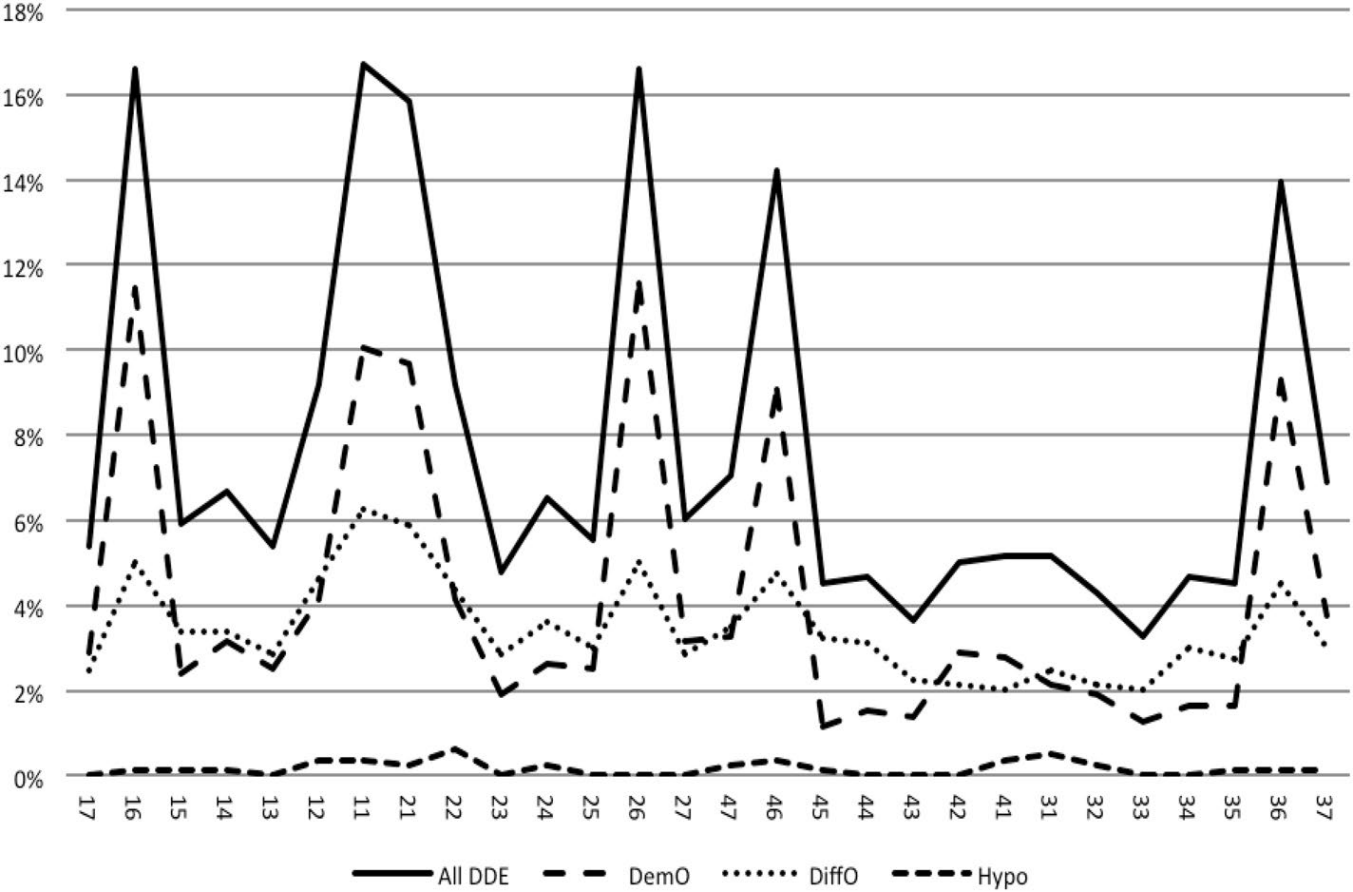
The frequency of demarcated opacities (DEO), diffuse opacities (DIO) and hypoplasias (Hypo) by tooth type

The most frequently affected teeth were the upper first molars and central incisors (16–17% of the total sample). They were the most affected teeth with both DEO and DIO. The mandibular canines were the least affected teeth. Hypoplasia was an uncommon defect (Fig. 2).

Chronological DDE, affecting FPM and incisors, was the most frequent defect, followed by generalised, non-chronological and local defects (Table 4).

**Table 4:** The distribution of local, chronological and non-chronological defects

	N (% total)	Affected teeth	N (% total)
Local	52 (6.5%)	Anterior teeth	42 (5.2%)
		Other teeth	10 (1.3%)
Chronological	119 (15%)	FPM and incisors (FP&I)	104 (13%)
		Other chronological	15 (2%)
Non-chronological	93 (12%)	Generalised	67 (8%)
		FPM&I plus 1–2 other teeth	18 (2%)
		< 4 teeth	8 (1%)

### Generalised DDE and MIH

Generalised DDE was seen in 8.4% of the total sample, when denominating non-chronological defects affecting more than four teeth as generalised. The mean number of affected teeth in this group was 15.2 (SD 7.0). Almost 25% of those with generalised DDE had both DEO and DIO. The remainder had either solely DIO or DEO, to a similar extent. Only one patient with generalised DDE had merely hypoplasia. The occurrence of generalised DDE in the different cohorts, as well as in the different clinics, was statistically significant.

When taking the clinical registrations and information from dental records into account, 12.2% of the total sample was judged to have MIH. There was no significant difference among the participating clinics, but the youngest cohort had a higher prevalence (17.1%) of MIH (Tables 2, 3).

### Local defects

The majority of the local defects were in the front teeth (incisors and canines) (Table 4). In eight cases, a single premolar was the only affected tooth.

### Aetiological factors

Traumatic injuries to the primary front teeth before 4 years of age raised the risk for DDE to the permanent successors (OR 2.3, CI 1.6–3.4). Injuries to 4–6-year-olds had no significant influence (OR 1.4, CI 0.8–2.3) (Table 5).

**Table 5:** The relation between traumatic injuries of the primary frontal teeth, before and after 3 year of age, and developmental defects of the enamel (DDE) in the permanent frontal teeth

	Trauma	No trauma	Total	
0–3 years				
DDE front teeth	52	121	173	
No DDE front teeth	100	523	623	
Total	152	644	796	OR 2.3, CI 1.6–3.4
4–6 years				
Front teeth DDE	23	150	173	
No front teeth DDE	62	561	561	
Total	85	711	796	OR 1.4, CI 0.8–2.3

One-third of the DDE group (34%) used drinking water from a well. Four of them reported raised fluoride content in the water. The prevalence of diffuse opacities was slightly higher (p = 0.212) in this group, compare to those in the DDE-group with municipal water.

Reported health problems in early childhood did not raise the prevalence of DDE.

Twelve percent reported possible heredity.

### Dental fear and anxiety

The presence of DDE did not lead to dental fear and anxiety. The DDE group scored 19.7 and the non-DDE group scored 20.0 in the CFSS-DS instrument (p = 0.54).

### Aesthetic considerations

**Fig. 3:**
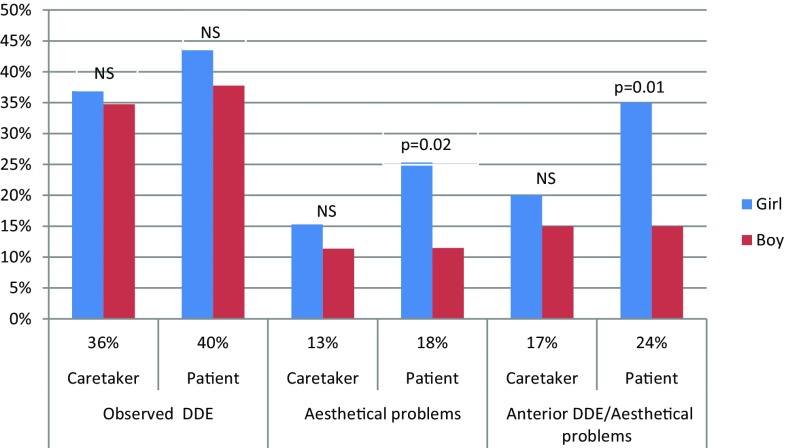
The observation of developmental defects of the enamel (DDE) by the patients and the caretakers in relation to the gender of the patient

**Fig. 4:**
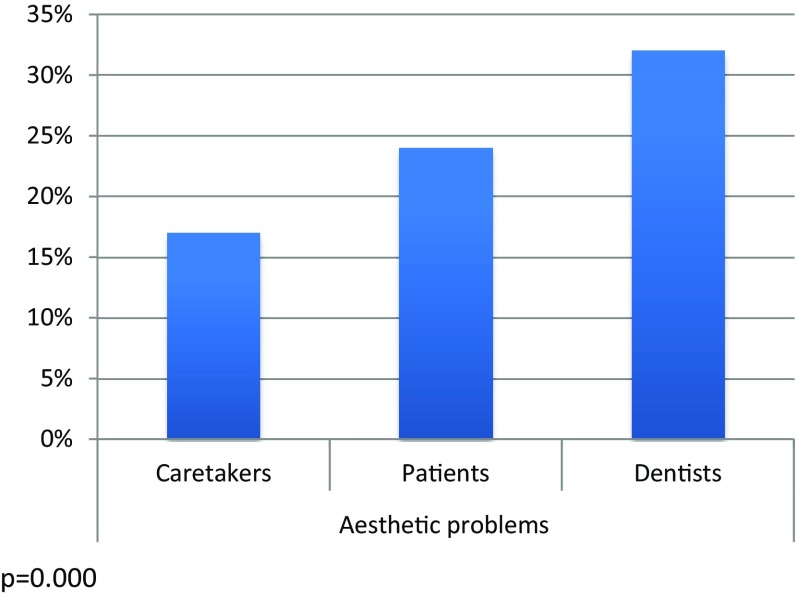
The caretakers, the patients’ and the dentists’ judgments of aesthetic problems in cases with frontal developmental defects of the enamel (DDE)

Approximately 40% of the caretakers and children/adolescents had observed the DDE, with about half of them regarding the defects as an aesthetic problem. Those with anterior DDE were more concerned. The children/adolescents with frontal DDE were more troubled than their caretakers, with girls and their caretakers more concerned than boys and their caretakers. The paediatric dentists were more concerned with the aesthetic problems than the patients and their caretakers (Figs. 3, 4).

## Discussion

The frequency of developmental defects of the enamel (DDE) was comparable with an earlier cohort study in the present region. The mixture of different types of DDE within an individual was frequent, making diagnoses based on the clinical appearance of DDE hazardous and doubtful. There is also a risk of over-diagnosing MIH, as the first permanent molars and upper incisors were the most frequently affected teeth of demarcated, as well as diffuse opacities, especially before it is possible to inspect premolars and second permanent molars. Furthermore, the paediatric dentists were more concerned about the appearance of frontal DDE than the patients and their caretakers.

One-third (33.2%) of the examined population had DDE of any type, with demarcated opacities (DEO) as the most frequent defect. Compared to other studies using the mDDE-index (Clarkson and O’Mullane 1992; Seow 2011; Robies et al. 2013), the DDE prevalence was lower in the present study. The absence of malnutrition and well-functioning healthcare during early childhood, may have contributed to a lower DDE prevalence.

In the total sample, DDE was commoner in the 15-year-old cohort, possibly because not all permanent teeth had erupted in the younger cohort and some DDE in the older cohort had faded, e.g., in mild fluorosis, just a very superficial layer of the enamel is affected and might easily be worn away (Wong et al. 2016).

In accordance with other studies, DEO was the dominating type of defect (Clarkson and O’Mullane 1992; Robies et al. 2013). However, this study also showed that DEO in combination with other defects, mostly diffuse opacities (DIO), was common, e.g., in a number of cases with verified MIH, DIO in the second permanent molars and/or premolars was registered. A possible explanation could be that the patients were affected by MIH as well as by mild fluorosis. Another suggestion is that there could also be an individual susceptibility to enamel developmental defects. The mixture of different types of DDE makes establishing the cause and diagnosis problematic.

Only 4% of the study group had any teeth with hypoplasia comparable with the findings from Ireland (Clarkson and O’Mullane 1992), while the corresponding figure in Australia was 17% (Seow 2011). A suspicion is that enamel disintegration may have been registered as hypoplasias. In addition, all DDE was formerly denominated hypoplasias, and that term may have a tendency to survive.

In accordance with other studies (Seow 2011; Robies et al. 2013), the first permanent molars (FPM) and the maxillary central incisors (PCIm) were the most affected teeth. The present study showed that DEO and DIO are the most prevalent type of defects in these teeth.

MIH is the main reason for the elevated occurrence of DDE in PFM and PCI, but these teeth also seem to be the target teeth for other types of enamel disturbances. However, the raised prevalence of DIO in FPM is hard to explain. Possibly, the ameloblasts in teeth, mineralised at a very early age, are more susceptible to any disturbances. The estimated MIH prevalence was in concordance with other studies (Jälevik 2010). The prevalence in the youngest age group was higher (17.1%). A comparable prevalence of 18.4% in 8-year-olds was shown in a prior MIH prevalence study in this region (Jälevik et al. 2001). In preventing misdiagnosis as a result of disintegration, caries, or restorations, the 8-year-olds have been considered to be the best age for the recognition of MIH (Weerheim et al. 2003). However, there may be a risk of over-diagnosing when the defects in FPM are mild, and when the second molars, premolars, and canines cannot be judged, because they are not erupted. When searching for the aetiology of MIH, a correct diagnosis is of utmost importance. The frequent occurrence of DIO in FPM could also lead to an over-diagnosis of MIH, especially when only target teeth are examined.

DDE in the permanent incisors can be caused by traumatic injuries to the primary incisors. In accordance with other research (Andreassen et al. 1971), the present study showed a significant risk for DDE in the permanent successor in cases of trauma in early childhood. Notably, the risk for DDE diminished considerably in cases of injuries to 4 to 6-year-olds.

Few children (1%) had an affected, solitary premolar that might have been caused by an infected primary molar.

Almost one in ten had generalised, non-chronological defects, which were either genetic or due to non-genetic, long-standing environmental influences (Espelid et al. 2017). The prevalence of amelogenesis imperfecta (AI) was 1/4000 in this region in 1985 (Sundell et al. 1985). It is not likely that the prevalence of AI has dramatically increased. Long-standing environmental influences remains to be suspected. Mild fluorosis, caused by the ingestion of toothpaste or other fluoride supplements is plausible (Seow 2011), as there was no significant relation between drinking water from a well and generalised non-chronological defects. However, this does not explain the frequent, generalised, non-chronological defects, solely with DEO.

The CFSS-DS instrument results, scoring dental fear and anxiety, was very low in the present study-group, indicating good dental healthcare from an early age. In contrast to a previous study in this geographic region (Jälevik and Klingberg 2002), not even those with DDE showed raised CFSS-DS scores. These improvements may depend on better knowledge of caring for children with DDE, but also the fact that the former study was dealing only with severe MIH.

Frontal DDE led to aesthetic problems in every forth case. Thus, the majority did not worry about the deviant appearance of their front teeth. As expected, girls were significantly more troubled than boys. These findings are in accordance with Sujak et al. (2004). Notably, the dentists were significantly more concerned than the patients. Consequently, the dentists must be careful not to transmit their opinion concerning appearance to the patients.

## Conclusions

The prevalence of any type of DDE was 33.2%, somewhat lower than other comparable studies. The 15-year age cohort and the most rural clinic had a significantly higher prevalence.

The mixture of different types of developmental defects in enamel (DDE), within one individual, was frequent, making diagnoses based on the clinical appearance of DDE hazardous and doubtful.

There was a significant risk for DDE in the permanent successor in cases of trauma before 4 years of age.

Reported health problems in early childhood did not raise the prevalence of DDE.

The presence of DDE did not lead to a raised level of dental fear and anxiety.

The paediatric dentists were more concerned about the appearance of anterior DDE than the patients and their caretakers.
